# Characteristics and use of urban health indicator tools by municipal built environment policy and decision-makers: a systematic review protocol

**DOI:** 10.1186/s13643-017-0406-x

**Published:** 2017-01-13

**Authors:** Helen Pineo, Ketevan Glonti, Harry Rutter, Nicole Zimmermann, Paul Wilkinson, Michael Davies

**Affiliations:** 1 Institute of Environmental Design and Engineering, Bartlett School of Environment, Energy and Resources, University College London, Central House, 14 Upper Woburn Place, London, WC1H 0NN UK; 2 ECOHOST–The Centre for Health and Social Change, London School of Hygiene and Tropical Medicine, 15-17 Tavistock Place, London, WC1H 9SH UK; 3 Department of Social and Environmental Health Research, London School of Hygiene and Tropical Medicine, Keppel Street, London, WC1E 7HT UK

**Keywords:** Urban metrics, Built environment, Indicator, Indices, Policy, Urban health, Evidence, Urban, Social determinants of health

## Abstract

**Background:**

There is wide agreement that there is a lack of attention to health in municipal environmental policy-making, such as urban planning and regeneration. Explanations for this include differing professional norms between health and urban environment professionals, system complexity and limited evidence for causality between attributes of the built environment and health outcomes. Data from urban health indicator (UHI) tools are potentially a valuable form of evidence for local government policy and decision-makers. Although many UHI tools have been specifically developed to inform policy, there is poor understanding of how they are used. This study aims to identify the nature and characteristics of UHI tools and their use by municipal built environment policy and decision-makers.

**Methods:**

Health and social sciences databases (ASSIA, Campbell Library, EMBASE, MEDLINE, Scopus, Social Policy and Practice and Web of Science Core Collection) will be searched for studies using UHI tools alongside hand-searching of key journals and citation searches of included studies. Advanced searches of practitioner websites and Google will also be used to find grey literature. Search results will be screened for UHI tools, and for studies which report on or evaluate the use of such tools. Data about UHI tools will be extracted to compile a census and taxonomy of existing tools based on their specific characteristics and purpose. In addition, qualitative and quantitative studies about the use of these tools will be appraised using quality appraisal tools produced by the UK National Institute for Health and Care Excellence (NICE) and synthesised in order to gain insight into the perceptions, value and use of UHI tools in the municipal built environment policy and decision-making process. This review is not registered with PROSPERO.

**Discussion:**

This systematic review focuses specifically on UHI tools that assess the physical environment’s impact on health (such as transport, housing, air quality and greenspace). This study will help indicator producers understand whether this form of evidence is of value to built environment policy and decision-makers and how such tools should be tailored for this audience.

**Systematic review registration:**

N/A.

**Electronic supplementary material:**

The online version of this article (doi:10.1186/s13643-017-0406-x) contains supplementary material, which is available to authorized users.

## Background

The impact of the urban environment on health outcomes has been widely documented [1–3], but there remains a lack of attention to health in municipal environmental policy-making, such as urban planning and regeneration [4, 5]. Researchers point to several potential explanations for the perceived lack of action from policy-makers, including differing professional norms, system complexity and limited evidence for causality between attributes of the built environment and health outcomes [2, 5, 6]. Urban health indicators are a resource which could help overcome some of these challenges and inform evidence-based municipal policies on the social determinants of health. The WHO Healthy Cities projects and a variety of other research and policy programmes have used data from urban health indicators (UHIs) as evidence to inform strategies and policies [7–10].

The use of evidence to inform policy and decision-making in municipal services that impact the wider determinants of health is an under-researched area [11, 12]. Recent studies have found that built environment professionals value data and expertise about the local context over academic evidence [12, 13]. Many indicator tools claim to be evidence-based and are underpinned by research evidence and expert involvement. Therefore urban health indicators may be one route through which research evidence informs policy, if they are used in the policy-making process [10]. Several recent reviews of urban health indicators have focused on describing the nature of indicators and challenges with their development—none have evaluated studies on their use by policy and decision-makers [7, 9, 14, 15]. It is therefore unclear whether this form of evidence is reaching its intended audience and aiding with the process of policy and decision-making.

Urban health and environmental health indicators have traditionally focused on health outcomes and environmental risks such as pollution [16]. The rise of non-communicable diseases and links to urban form and development patterns have broadened the scope and purpose of urban health indicators [17]. There have been recent attempts to conceptualise the interconnections and complex relations between the environmental determinants of health and other related policy objectives such as liveability, quality of life, wellbeing and sustainability [2, 5, 7]. A number of recent indicator tools are attempting to make these relations more explicit with the intention of informing and shaping policies and decisions that meet these aligned objectives.^1^ These tools provide indicators on multiple aspects of the urban environment simultaneously, therefore recognising and highlighting the complexity of the system. Examples of urban health indicators include access to recreational facilities (measured by the San Francisco Indicator Project as ‘proportion of population within 1/4 mile of a public recreation facility’) and access to public transport (measured by the Community Indicators Victoria tool as ‘average distance to nearest public transport stop including tram, bus and train stops (kilometers)’. It is these tools which could be of most use to urban planners and other municipal built environment policy and decision-makers who need to balance multiple sustainability objectives with competing interests, such as economic viability and local acceptability [18]. Although researchers argue that indicators can be used to help policy-makers understand and respond to complex systems, this claim is not supported by research evidence on the use of indicators for this purpose [9, 19].

## Defining urban health indicator tools

A number of concepts were explored in the health and urban planning literature to develop a definition for ‘urban health indicator tool’ for this review. Galea and Vlahov define urban health as ‘the determinants of health and diseases in urban areas and with the urban context itself as the exposure of interest’ [1]. There are numerous definitions for an ‘indicator’ which vary by the policy fields for which they were created. Kotval describes an indicator as ‘a measure or a set of measures that describes a complex social, economic or physical reality’ [20]. While Pencheon refers to indicators as ‘succinct measures that aim to describe as much about a system as possible in as few points as possible’ [21]. This review defines an ‘urban health indicator tool’ as a collection of summary measures about the physical urban environment’s contribution to human health and wellbeing. This definition broadens the scope of ‘health’ to include related concepts of quality of life, liveability and wellbeing.

## Aims/objectives

This study aims to investigate the nature and characteristics of urban health indicator tools and their perceived value and use by municipal built environment policy and decision-makers. The specific objectives are the following:

1. To create a census and taxonomy of urban health indicator tools
2. To understand how UHI tools are used in the policy and decision-making process
3. To explore the perceived impact of UHI tools on policy and decision-making
4. To investigate the value of UHI tools in relation to simplifying, representing or addressing complex systems

## Method

This systematic review will include studies and grey literature to identify UHI tools and investigate their use by policy and decision-makers. The PRISMA-P guidelines have been followed, and the checklist is available in Additional file 1. The protocol was developed iteratively following a scoping review of relevant studies and urban health indicator tools. It was informed by similar social sciences systematic reviews regarding the relevant population of policy-makers [13, 22]. Draft iterations of the protocol were shared with an advisory group of senior researchers across health, policy and built environment fields and subsequently improved. This review is not registered with PROSPERO.

## Eligibility criteria

There are two parts to this systematic review, with each part having distinct criteria. The first part (part A) is a census of urban health indicator tools. Any UHI tool referred to in peer-reviewed or grey literature documents will be eligible for inclusion if it meets the definition of an urban health indicator tool outlined above and is published in English. Non-English language publications are excluded due to resource constraints and this is recognised as a limitation in the review which will be considered during analysis and reporting of results. UHI tools which only regard one aspect of the physical urban environment (such as air quality) are too narrow to meet the definition provided in this protocol and will therefore be excluded.

The second part of the review (part B) relates to studies about the use of UHI tools and includes any study design (including case studies). Studies will be included if they meet the following criteria:

- Reports substantive data on views, attitudes or knowledge about the use of an urban health indicator tool in the policy-making or decision-making process, or about the implementation of specific policies, interventions or programmes informed by these (modified from [13])
- Includes policy and/or decision-makers from one of the following policy fields in local government: housing, transport, urban planning and regeneration
- Reports qualitative or quantitative data
- Published in English

Studies reported in any country will be included initially. It may be necessary to limit studies to those that are similar to a UK context if the cultures of practise appear to be sufficiently different. There are no date restrictions.

## Information sources

Two University College London (UCL) librarians specialising in systematic reviews have helped to identify the search strategy and appropriate bibliographic databases for the review. The following health and social sciences databases will be searched: Applied Social Sciences Index and Abstracts (ASSIA), Campbell Library, EMBASE, MEDLINE, Scopus, Social Policy and Practice and Web of Science Core Collection (includes Social Sciences Citation Index). In addition, a hand-search of the following key journals will be conducted: Annual Review of Public Health, Social Science and Medicine, BMC Public Health and Social Indicators Research. Citation searches of included studies will be performed. Following advice from the UCL librarian, Google Advanced Search will be used to systematically search using specified search terms, including a focus on the following practitioner websites: Town and Country Planning Association (UK), Royal Town Planning Institute (UK), Planning Institute of Australia, American Planning Association, Built Environment and Public Health Clearinghouse and the World Health Organization Europe.

## Search strategy

The search terms and MeSH subject headings were identified through a scoping study of urban health indicator publications. Search terms and indicators identified in similar reviews were also examined and trialled to identify the key terms [7, 9, 15]. The key terms for the inclusion criteria were related to the urban environment (e.g. urban, metropolitan, city, environment, neighbourhood, community), health and related concepts (e.g. determinant, public, health, wellbeing, wellness, quality, liveability) and indicator terms (e.g. benchmark, tool, indicator, index, indices, measure, metric, profile, assessment, score, standard). In Scopus, Web of Science and Ovid (EMBASE and MEDLINE), subject areas were limited to refine results (e.g. subjects such as pharmacology and dentistry were excluded). Boolean and adjacency operators were also used to construct the search and refine results. An example of the Ovid MEDLINE search is included in Additional file 2.

## Data management and selection

All records will be imported into EppiReviewer, specialist systematic review software, and duplicates will be removed. A second reviewer (KG) will screen a randomly selected sample of 10% of titles and abstracts. Inter-rater agreement percentages will be reported for both screening stages. Conflicts will be discussed and agreed upon with a third researcher (HR). Records will be removed at this stage if they do not meet the inclusion criteria for part A (i.e. they do not mention an urban health indicator tool or are not published in English). The second reviewer will screen a randomly selected sample of 10% of full papers. This pool of studies will include records for part A and part B. Full papers will be screened simultaneously for the inclusion criteria in part A and part B. The result will be a set of included urban health indicator tools and a set of included studies about the use of these tools.

## Data extraction

For each included urban health indicator tool (identified in part A), we will use the provided references or a Google search to determine if there is a stand-alone website and/or further documentation about the indicator and its methodology. The data will be extracted from the indicator tool website or other documentation by the indicator tool producer where possible. The preference is to find information about the indicator tool directly from its producer rather than third-party summaries or evaluations. If the information is only available through the included study, then the data will be extracted from there. The information source will be logged as part of the data extraction. A draft data extraction form in Excel has been developed (see Additional file 3). The form was iteratively developed using information from the scoping review. The data extraction will include the following categories (developed from the scoping review):

- Scale—At what scales can the system be applied or measured? (e.g. neighbourhood or city)
- Geography—Which areas can this system be applied in (e.g. specific cities or nations)?
- Scope—What aspects are analysed (e.g. built environment, health outcomes, demographics)?
- Producer—Which organisation developed the system? What type of organisation?
- Funders—Which organisations funded the indicator system?
- Purpose—What is the stated purpose? (e.g. research and/or informing policy)
- Methodology—Is there a published methodology and what are its characteristics?
- Evidence base—Does the methodology refer to evidence which was used to inform the system? What is the nature of this evidence?
- Weighting—Is there a weighting system and what are its characteristics?
- Complexity—Does the methodology refer to complexity and, if so, in what context?
- Uncertainty—Does the methodology refer to uncertainty and, if so, in what context?
- Maps—Is there an option to view the data on maps?
- Publication date—When was the system published?
- Source—Where was this information found?
- Indicators—Which indicators are reported?

Studies that meet inclusion criteria for part B will be included in a narrative synthesis. The following data will be extracted in an Excel sheet (see Additional file 4) for each study included in part B (informed by [13, 22]):

- Author, year
- Country
- Year that study was carried out
- Urban health indicator tool being evaluated
- Policy field
- Research parameters
- Data collection methods
- Population and sample selection
- Outcomes
- Analysis methods
- Limitations
- Funding source
- Conflicts of interest

A data extraction form has been created in Excel and any quantitative data will be analysed using Excel. Qualitative data will be synthesised using NVivo qualitative data analysis Software (QSR International Pty Ltd., Version 11, 2015). Data will be coded using an open code set. These will be updated in an iterative process as new factors regarding the perceptions and use of urban health indicator tools are identified.

## Quality appraisal

Studies about the use of UHI tools (part B) will be appraised using the quality appraisal tools for qualitative and quantitative studies produced by the UK National Institute for Health and Care Excellence (NICE) [23]. A modified version of the NICE tool for quantitative studies reporting correlations and associations will be used as there is no suitable version for study designs reporting quantitative data on participants’ perceptions. For qualitative studies, the NICE appraisal checklist includes assessment of the following: theoretical approach, study design, data collection, trustworthiness, analysis and ethics. For quantitative studies, the following topics are appraised: population, method of selection, outcomes, analyses and summary. A copy of the completed checklists will be published with the review results as an additional file.

## Data synthesis

There will be two components to the data synthesis. Data about the UHI tools will be analysed to create a taxonomy of the types of tools available to municipal built environment policy and decision-makers. This will include quantitative analysis of the physical urban environment features being measured, such as the percentage of UHI tools measuring particular features. The analysis will also assess the proportion of tools operating in particular geographic scales, the number of tools published each year, the proportion of UHI tools which mention complexity and the proportion of tools developed by different organisation types. The narrative synthesis of qualitative data from part B of this review will identify any recurrent themes across the studies regarding the perceptions and value of urban health indicator tools by policy and decision-makers.

## Discussion

Many urban health indicator tools are created with the goal of informing policy- and decision-makers who influence the social determinants of health in urban environments. However, there is little clarity about what type of evidence this audience uses and whether urban health indicator tools form part of their evidence toolbox. This review focuses specifically on municipal built environment policy and decision-makers and their perceptions of UHI tools. The findings will be of value to UHI tool producers who wish to target their tools to this audience with the aim of improving the health impact of urban environments.

Complexity is emphasised as a key challenge in relation to policy-making for health and the built environment, in response to which indicators have been promoted as a solution. This review will seek to understand whether and how UHI tools aim to address the complexity of the systems they measure. An initial scoping review showed that this topic is not widely addressed in indicator methodology documents. This review will seek to understand whether UHI tools are perceived as assisting with complexity in the policy and decision-making process. However, it is recognised that studies identified for this review may not address this topic. Therefore, this review will also help to establish the current research evidence supporting the claim that indicators are a tool to support policy and decision-makers operating in this complex system.

## Additional files

Additional file 1: PRISMA-P Checklist. Completed PRISMA-P Checklist. (DOCX 37 kb)
Additional file 2: Title of data: MEDLINE search. MEDLINE (Ovid) search strategy. (DOCX 12 kb)
Additional file 3: Data extraction form part A. Data extraction form part A. (XLS 33 kb)
Additional file 4: Data extraction form part B. Data extraction form part B. (XLS 22 kb)

## Abbreviations

ASSIA: Applied Social Sciences Index and Abstracts
MeSH: Medical Subject Headings
NICE: National Institute for Health and Care Excellence
PRISMA-P: Preferred Reporting Items for Systematic review and Meta-Analysis Protocols
UCL: University College London
UHI: Urban health indicator

## Footnotes

1 For example, see the San Francisco Indicator Project developed by the SF Department of Public Health and the City and County of San Francisco (http://www.sfindicatorproject.org/ accessed 29 Mar 2016) and the Community Indicators Victoria tool developed by McCaughey Centre, VicHealth and the University of Melbourne (http://www.communityindicators.net.au/ accessed 17 Feb 2016).
