# Supplementary material for: Characteristics and use of urban health indicator tools by municipal built environment policy and decision-makers: a systematic review protocol
Source: Syst Rev. 2017 Jan 13;6:2. doi: 10.1186/s13643-017-0406-x (PMC5237355; doi:10.1186/s13643-017-0406-x)
Supplement: Additional file 1: — PRISMA-P Checklist. Completed PRISMA-P Checklist. (DOCX 37 kb) [file 13643_2017_406_MOESM1_ESM.docx]

**PRISMA-P (Preferred Reporting Items for Systematic review and Meta-Analysis Protocols) 2015 checklist: recommended items to address in a systematic review protocol***

| Section and topic | Item No | Checklist item | Information from protocol |
| --- | --- | --- | --- |
| ADMINISTRATIVE INFORMATION | | |  |
| Title: |  |  |  |
| Identification | 1a | Identify the report as a protocol of a systematic review | Protocol titled ‘Characteristics and use of urban health indicator tools by municipal built environment policy and decision-makers: a systematic review protocol’ |
| Update | 1b | If the protocol is for an update of a previous systematic review, identify as such | N/A |
| Registration | 2 | If registered, provide the name of the registry (such as PROSPERO) and registration number | N/A |
| Authors: |  |  |  |
| Contact | 3a | Provide name, institutional affiliation, e-mail address of all protocol authors; provide physical mailing address of corresponding author | Helen Pineo^1^, Ketevan Glonti^2^, Harry Rutter^2^, Nicole Zimmermann^1^, Paul Wilkinson^3^, Michael Davies^1^  ^1^Institute of Environmental Design and Engineering, Bartlett School of Environment, Energy and Resources, University College London, Central House, 14 Upper Woburn Place, London, WC1H 0NN, helen.pineo.15@ucl.ac.uk, n.zimmermann@ucl.ac.uk, michael.davies@ucl.ac.uk  ^2^ECOHOST – The Centre for Health and Social Change, London School of Hygiene and Tropical Medicine, 15-17 Tavistock Place, London, WC1H 9SH, ketevan.glonti@lshtm.ac.uk, harry.rutter@lshtm.ac.uk  ^3^Department of Social and Environmental Health Research, London School of Hygiene and Tropical Medicine, Keppel Street, London, WC1E 7HT, paul.wilkinson@lshtm.ac.uk |
| Contributions | 3b | Describe contributions of protocol authors and identify the guarantor of the review | NZ, KG, HR, PW, MD advised on the study design. HP designed the study, performed the searches, imported results into EppiReviewer, performed deduplication and wrote the protocol. HP will screen records, perform quality appraisal, extract data and analyse data. KG will screen a randomly sampled selection of titles, abstracts and full papers. All authors read and approved the protocol. All authors will read the final systematic review paper. The guarantor of the review is Helen Pineo. |
| Amendments | 4 | If the protocol represents an amendment of a previously completed or published protocol, identify as such and list changes; otherwise, state plan for documenting important protocol amendments | The protocol was developed iteratively during the scoping review process. Any changes to the final published version will be noted and appended to the systematic review publication. |
| Support: |  |  |  |
| Sources | 5a | Indicate sources of financial or other support for the review | The Building Research Establishment Ltd. (BRE) is funding the review via HP’s PhD sponsorship. |
| Sponsor | 5b | Provide name for the review funder and/or sponsor | Same as above. |
| Role of sponsor or funder | 5c | Describe roles of funder(s), sponsor(s), and/or institution(s), if any, in developing the protocol | The funder was not involved in developing the protocol apart from HP’s role as a part-time PhD student employed by the BRE. |
| INTRODUCTION | | |  |
| Rationale | 6 | Describe the rationale for the review in the context of what is already known | Previous reviews of urban metrics and urban health indicators (UHIs) have not evaluated the use of these tools by built environment policy and decision-makers. It is not clear whether this policy audience is using UHIs and how they are perceived and valued in professional practice. See ‘background’ for more information. |
| Objectives | 7 | Provide an explicit statement of the question(s) the review will address with reference to participants, interventions, comparators, and outcomes (PICO) | This study aims to investigate the nature and characteristics of urban health indicator tools and their perceived value and use by municipal built environment policy and decision-making. The specific objectives are:   1. To create a census and taxonomy of urban health indicator tools. 2. To understand how UHI tools are used in the policy and decision-making process 3. To explore the perceived impact of UHI tools on policy and decision-making. 4. To investigate the value of UHI tools in relation to simplifying, representing or addressing complex systems.   Population: Built environment policy and decision-makers in local government: urban planners, transport planners, housing officers, regeneration officers and elected members in these policy areas.  Intervention: Use of urban health indicators in the policy and decision-making process  Comparison: N/A  Outcome: views, attitudes or knowledge about the use of an urban health indicator tool in the policy-making or decision-making process, or about the implementation of specific policies, interventions or programmes informed by UHI tools |
| METHODS | | |  |
| Eligibility criteria | 8 | Specify the study characteristics (such as PICO, study design, setting, time frame) and report characteristics (such as years considered, language, publication status) to be used as criteria for eligibility for the review | There are two parts to this systematic review, with each part having distinct criteria. The first part (Part A) aims to establish a census of urban health indicator tools. Any reference to UHI tools in peer-reviewed or grey literature documents will be eligible. The included UHI tools for Part A must meet the definition of an urban health indicator tool outlined above and be published in English. UHI tools which only regard one aspect of the physical urban environment (such as air quality) are too narrow to meet the definition provided in this protocol and will therefore be excluded.  The second part of the review (Part B) relates to studies about the use of UHI tools and includes any study design (including case studies). The studies are included if they meet the following criteria:   - Reports substantive data on views, attitudes or knowledge about the use of an urban health indicator tool in the policy-making or decision-making process, or about the implementation of specific policies, interventions or programmes informed by these (modified from [13]). - Includes policy and/or decision-makers from one of the following policy fields in local government: housing; transport; urban planning and regeneration. - Reports qualitative or quantitative data. - Published in English.   Studies reported in any country will be included initially. It may be necessary to limit studies to those that are similar to a UK context if the cultures of practice appear to be sufficiently different. There are no date restrictions. |
| Information sources | 9 | Describe all intended information sources (such as electronic databases, contact with study authors, trial registers or other grey literature sources) with planned dates of coverage | Two University College London (UCL) librarians specialising in systematic reviews have helped to identify the search strategy and appropriate bibliographic databases for the review (RM and TV). The following health and social sciences databases will be searched: Applied Social Sciences Index and Abstracts (ASSIA), Campbell Library, EMBASE, MEDLINE, Scopus, Social Policy and Practice, and Web of Science Core Collection (includes Social Sciences Citation Index). In addition, a hand-search of the following key journals will be conducted: Annual Review of Public Health, Social Science and Medicine, BMC Public Health, and Social Indicators Research. Citation searches of included studies will be performed. Following advice from the UCL librarian, Advanced Google Searches will be used to systematically search practitioner websites and Google using specified search terms. In addition to two focused Google searches (using multiple terms and Boolean operators), the following specific websites were explored through Advanced Google searches: Town and Country Planning Association (UK), Royal Town Planning Institute (UK), Planning Institute of Australia, American Planning Association, Built Environment and Public Health Clearinghouse, World Health Organization Europe. There are no date limitations beyond those imposed by the bibliographic databases. |
| Search strategy | 10 | Present draft of search strategy to be used for at least one electronic database, including planned limits, such that it could be repeated | Medline Ovid search:  1. city planning/ or environment design/ or urban renewal/  2. Urban Health/ or Urban Population/  3. (Urban or Metropolitan or City or Cities or Environment* or Neighbourhood or Neighborhood or Communit*).ti.  4. Cities/  5. 1 or 2 or 3 or 4  6. ((Determinant* or Public or Health* or Wellbeing or well being or Quality of life or Liveab* or Livab*) adj2 (Benchmark* or Tool* or Indicator* or Index* or Indices or Measure* or Metric* or Profile* or Assessment* or Score* or Standard*)).m_titl.  7. ((Determinant* or Public or Health* or Wellbeing or well being or Quality of life or Liveab* or Livab*) adj2 (Benchmark* or Tool* or Indicator* or Index* or Indices or Measure* or Metric* or Profile* or Assessment* or Score* or Standard*)).tw.  8. exp Health Status/  9. exp Health Status Indicators/  10. 8 or 9  11. 5 and 7 and 10  12. 5 and 6  13. 11 or 12  14. limit 13 to english language  15. exp animals/ not humans/  16. 14 not 15 |
| Study records: |  |  |  |
| Data management | 11a | Describe the mechanism(s) that will be used to manage records  and data throughout the review | EppiReviewer software will be used to manage all records and perform screening. Microsoft Excel will be used for data extraction and quantitative data analysis (if applicable). Qualitative data will be synthesised using NVivo qualitative data analysis Software; QSR International Pty Ltd. Version 11, 2015. |
| Selection process | 11b | State the process that will be used for selecting studies (such as two independent reviewers) through each phase of the review (that is, screening, eligibility and inclusion in meta-analysis) | All records will be imported into EppiReviewer, specialist systematic review software, and duplicates will be removed. A second reviewer will screen a randomly selected sample of 10% of titles and abstracts. Inter-rater agreement percentages will be reported for both screening stages. Conflicts will be discussed and agreed upon with a third researcher. Records will be removed at this stage if they do not meet the inclusion criteria for Part A (i.e. they do not mention an urban health indicator tool or are not published in English). A second reviewer will screen a randomly selected sample 10% of full papers. This pool of studies will include records for Part A and Part B. Full papers will be screened simultaneously for the inclusion criteria in Part A and Part B. The result will be a set of included urban health indicator tools and a set of included studies about the use of these tools. |
| Data collection process | 11c | Describe planned method of extracting data from reports (such as piloting forms, done independently, in duplicate), any processes for obtaining and confirming data from investigators | Data will be extracted in two separate Excel forms (for Part A and B respectively) independently. |
| Data items | 12 | List and define all variables for which data will be sought (such as PICO items, funding sources), any pre-planned data assumptions and simplifications | For Part A – the census of Urban Health Indicator Tools the following will be sought:   - Scale – At what scales can the system be applied or measured? (e.g. neighbourhood or city) - Geography – Which areas can this system be applied in (e.g. specific cities or nations)? - Scope – What aspects are analysed (e.g. built environment, health outcomes, demographics)? - Producer – Which organisation developed the system? What type of organisation? - Funders – Which organisations funded the indicator system? - Purpose – What is the stated purpose? (e.g. research and/or informing policy) - Methodology – Is there a published methodology and what are its characteristics? - Evidence-base – Does the methodology refer to evidence which was used to inform the system? What is the nature of this evidence? - Weighting – Is there a weighting system and what are its characteristics? - Complexity – Does the methodology refer to complexity and, if so, in what context? - Uncertainty – Does the methodology refer to uncertainty and, if so, in what context? - Maps – Is there an option to view the data on maps? - Publication date – When was the system published? - Source – Where was this information found? - Indicators – Which indicators are reported?   For Part B the analysis of the use of UHI tools, the following will be extracted:   - author, year - country - year that study was carried out - urban health indicator tool being evaluated - policy field - research parameters - data collection methods - population and sample selection - outcomes - analysis methods - limitations - funding source - conflicts of interest |
| Outcomes and prioritization | 13 | List and define all outcomes for which data will be sought, including prioritization of main and additional outcomes, with rationale | A narrative synthesis of outcomes (substantive data on views, attitudes or knowledge about the use of an urban health indicator tool in the policy-making or decision-making process, or about the implementation of specific policies, interventions or programmes informed by these) will use open coding. |
| Risk of bias in individual studies | 14 | Describe anticipated methods for assessing risk of bias of individual studies, including whether this will be done at the outcome or study level, or both; state how this information will be used in data synthesis | Given the nature of this review, there will be no assessment of risk of bias. Bias will be addressed as a limitation of the included study types. |
| Data synthesis | 15a | Describe criteria under which study data will be quantitatively synthesised | If quantitative data about views, attitudes and knowledge are obtained, these will be reported. It is unlikely that data from more than one study will be combined (if this is possible, it will be done in Excel). |
|  | 15b | If data are appropriate for quantitative synthesis, describe planned summary measures, methods of handling data and methods of combining data from studies, including any planned exploration of consistency (such as I^2^, Kendall’s τ) | Any quantitative data would likely be responses to surveys and these are not likely to be consistent across studies (and therefore not possible to combine). |
|  | 15c | Describe any proposed additional analyses (such as sensitivity or subgroup analyses, meta-regression) | N/A |
|  | 15d | If quantitative synthesis is not appropriate, describe the type of summary planned | There will be two components to the data synthesis. Data about the UHI tools will be analysed to create a taxonomy of the types of tools available to municipal built environment policy and decision-makers. This will include analysis of the physical urban environment features being measured, the scale at which they are measured and other observations on the nature of these tools. The narrative synthesis of qualitative data from Part B of this review will identify any recurrent themes across the studies regarding the perceptions and value of urban health indicator tools by policy and decision-makers. The qualitative data reported as the outcomes will be synthesised using NVivo. Data will be coded using an open code set. These will be updated in an iterative process as new factors regarding the perceptions and use of urban health indicator tools are identified. |
| Meta-bias(es) | 16 | Specify any planned assessment of meta-bias(es) (such as publication bias across studies, selective reporting within studies) | N/A |
| Confidence in cumulative evidence | 17 | Describe how the strength of the body of evidence will be assessed (such as GRADE) | Studies about the use of UHI tools will be appraised independently using the quality appraisal tool produced by the UK National Institute for Health and Care Excellence (NICE). A copy of the completed checklists will be made available with the study results. There will not be an assessment of the confidence of cumulative evidence. |

*** It is strongly recommended that this checklist be read in conjunction with the PRISMA-P Explanation and Elaboration (cite when available) for important clarification on the items. Amendments to a review protocol should be tracked and dated. The copyright for PRISMA-P (including checklist) is held by the PRISMA-P Group and is distributed under a Creative Commons Attribution Licence 4.0.**

*From: Shamseer L, Moher D, Clarke M, Ghersi D, Liberati A, Petticrew M, Shekelle P, Stewart L, PRISMA-P Group. Preferred reporting items for systematic review and meta-analysis protocols (PRISMA-P) 2015: elaboration and explanation. BMJ. 2015 Jan 2;349(jan02 1):g7647.*
