# Supplementary material for: Characteristics and use of urban health indicator tools by municipal built environment policy and decision-makers: a systematic review protocol
Source: Syst Rev. 2017 Jan 13;6:2. doi: 10.1186/s13643-017-0406-x (PMC5237355; doi:10.1186/s13643-017-0406-x)
Supplement: Additional file 2: — Title of data: MEDLINE search. MEDLINE (Ovid) search strategy. (DOCX 12 kb) [file 13643_2017_406_MOESM2_ESM.docx]

**Medline (Ovid) search strategy**

1. city planning/ or environment design/ or urban renewal/

2. Urban Health/ or Urban Population/

3. (Urban or Metropolitan or City or Cities or Environment* or Neighbourhood or Neighborhood or Communit*).ti.

4. Cities/

5. 1 or 2 or 3 or 4

6. ((Determinant* or Public or Health* or Wellbeing or well being or Quality of life or Liveab* or Livab*) adj2 (Benchmark* or Tool* or Indicator* or Index* or Indices or Measure* or Metric* or Profile* or Assessment* or Score* or Standard*)).m_titl.

7. ((Determinant* or Public or Health* or Wellbeing or well being or Quality of life or Liveab* or Livab*) adj2 (Benchmark* or Tool* or Indicator* or Index* or Indices or Measure* or Metric* or Profile* or Assessment* or Score* or Standard*)).tw.

8. exp Health Status/

9. exp Health Status Indicators/

10. 8 or 9

11. 5 and 7 and 10

12. 5 and 6

13. 11 or 12

14. limit 13 to english language

15. exp animals/ not humans/

16. 14 not 15
